# Hybrid Palliation for Hypoplastic Left Heart Syndrome: Role of Echocardiography

**DOI:** 10.3390/children10061012

**Published:** 2023-06-04

**Authors:** Lilia Oreto, Paolo Guccione, Placido Gitto, Letteria Bruno, Rosanna Zanai, Nadia Grasso, Enrico Iannace, Concetta Zito, Scipione Carerj, Salvatore Agati

**Affiliations:** 1 Department of Clinical and Experimental Medicine, University of Messina, 98122 Messina, Italy; loreto@unime.it (L.O.); czito@unime.it (C.Z.); scarerj@unime.it (S.C.); 2 Mediterranean Pediatric Cardiology Center, Bambino Gesù Children’s Hospital, 98035 Taormina, Italy; paolo.guccione@opbg.net (P.G.); dgitto72@yahoo.it (P.G.); rosanna.zanai@virgilio.it (R.Z.); nadiagrasso.ng@gmail.com (N.G.); enrico.iannace@opbg.net (E.I.); 3 Department of Human Pathology in Adult and Developmental Age, University of Messina, 98122 Messina, Italy; lilli.bruno@tiscali.it

**Keywords:** hybrid palliation, echocardiography, hypoplastic left heart syndrome

## Abstract

Hypoplastic left heart syndrome is a spectrum of complex congenital cardiac defects. Although in borderline cases, biventricular repair is a viable option, in the majority of cases, univentricular palliation is the treatment of choice. Hybrid palliation can be a valid alternative to classic Norwood operation in the neonatal period, especially in selected cases such as high-risk patients or borderline left ventricles. Echocardiography is the main diagnostic modality in this pediatric population, from the fetal diagnosis to the subsequent surgical steps of palliative treatment. Hybrid palliation is performed after birth and is characterized by surgical banding of the pulmonary arteries along with transcatheter stenting of the ductus arteriosus. There are some peculiar aspects of cardiac imaging that characterize this type of palliation, and that should be considered in the different phases before and after the procedure. We aimed to review the current literature about the role of echocardiography in the management of patients with hypoplastic left heart undergoing hybrid palliation.

## 1. Introduction

Hypoplastic left heart syndrome (HLHS) is a spectrum of complex congenital cardiac defects in which the anatomy and physiology of the heart are profoundly altered. Diagnosis of HLHS is based exclusively on ultrasound imaging modalities, whose versatility offers high accuracy and widespread availability. Prenatal diagnosis is of paramount importance since it allows to program the most appropriate management of the newborn with HLHS in a tertiary care center. Then, echocardiography provides important information for planning the subsequently staged palliation, and it is essential to watchfully care for the patient afterward.

Although the classic surgical approach in the first stage is the Norwood operation, the hybrid strategy has become a valid alternative in the last 20 years [1,2]. Hybrid palliation is based on selective surgical banding of the pulmonary arteries and transcatheter stenting of the ductus arteriosus without the use of cardiopulmonary bypass and circulatory arrest. Being significantly less invasive compared to a high-risk neonatal surgery like the Norwood operation, the hybrid procedure has gained benefits in selected cases of high-risk neonates with very low birth weight or significant comorbidities [3]. Moreover, in the borderline left ventricle, hybrid palliation has the advantage of serving as a bridge to a decision in order to allow the borderline ventricle to grow enough to deal with a biventricular repair. Finally, hybrid palliation as a first operation has the advantage of leaving open different surgical pathways since it can be followed by a delayed Norwood, a comprehensive stage II operation, or a biventricular repair, depending on the case.

Clinical and surgical management of patients with HLHS is based largely on cardiac imaging, mainly echocardiography, aimed to evaluate different targets in every different phase, from fetal life to various surgical steps [4]. In the case of hybrid palliation, echocardiography is needed to evaluate some peculiar aspects that deserve special consideration and that are crucial for the whole management, from the initial candidacy to the procedure, through the interstage, to subsequent surgical steps. Echocardiography is the most versatile imaging modality, which is virtually universally available at the bedside, providing a comprehensive evaluation of cardiovascular anatomy and function without any risk or discomfort for the patient. On the other hand, ultrasound imaging has a limited role in hemodynamic assessment, where cardiac catheterization and magnetic resonance provide deeper information at the expense of the necessary use of sedation or general anesthesia in the neonatal and pediatric age. Moreover, post-surgical echocardiography has some limitations, not only due to repeat sternotomy with a worse acoustic window but also because prosthetic tissue and suture lines may alter the profile of native structures.

Our aim was to review the current knowledge about the role of echocardiography in the management of hybrid palliation for HLHS (Table 1).

## 2. Fetal Life

**Figure 1:**
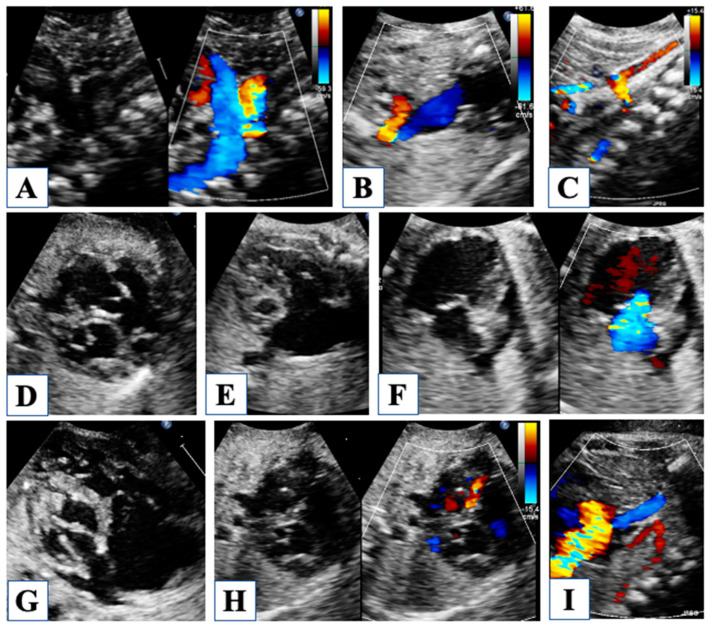
Fetal echocardiography. Reverse flow in the aortic arch is shown from a long-axis view of the aortic arch and ductal arch (panel (**A**)) and from a three-vessel view (panel (**B**)). Panel (**C**) shows severe hypoplasia of all the segments of the aortic arch with retrograde flow. Four-chamber view in panel (**D**) shows mitral valve stenosis with hypoplastic left ventricle, which does not form cardiac apex. In the four-chamber view in panel (**E**) mitral valve is atretic, and the left ventricle is a virtual chamber. Tricuspid valve with thickened leaflets and severe regurgitation is shown in Panel (**F**). In panel (**G**), the mitral valve is highly dysplastic, with a very small opening in diastole. Panel (**H**) shows a left-to-right shunt through the foramen ovale with inverted movement of the membrane. A vertical vein draining into the left atrium is shown in Panel (**I**).

There is great interest in prenatal diagnosis of HLHS, and the argument has been accurately discussed [21,22,23]. Although in the first trimester, the anatomy of the heart may not be well defined, as the only clue can be a hyperechogenic endocardial border of a near-normal left ventricle, in the second trimester, the anatomy appears extremely abnormal [23]. The four-chamber standard views first raise suspicion of HLHS by the observation of an underdeveloped left ventricle (Figure 1).

Then, a careful, thorough study is required for the evaluation of the hallmarks of HLHS: mitral atresia or stenosis, aortic atresia or stenosis, and hypoplastic aortic arch. The three major anatomic variants include (1) mitral atresia and aortic atresia, (2) mitral stenosis and aortic atresia, (3) mitral stenosis and aortic stenosis. For instance, the grade of development of the LV cavity and ascending aorta is proportional to the severity of mitral and aortic stenosis, respectively, depending on the amount of forward blood flow. Due to the inability of the left ventricle to provide adequate cardiac output, systemic circulation is dependent on the right ventricle through the ductus arteriosus, while the pulmonary venous return is redirected to the right chambers through the foramen ovale. In addition to a segmental and sequential anatomic definition of the fetal heart, special consideration is needed for the foramen ovale and for the ductus arteriosus since systemic circulation in this setting is provided exclusively through these fetal communications.

A small amount of fetal cardiac output is directed to the lungs; even though small, it is crucial for the proper development of the pulmonary vasculature [21]. Consequently, pulmonary venous flow to the left atrium is poor during fetal life, yet it must entirely stream to the right atrium since it has no other way to egress in the presence of mitral and/or aortic atresia. For this reason, blood flows through the foramen ovale is opposed to normal, being entirely or mainly left to right (Figure 1). An unrestrictive shunting across a wide-open foramen ovale is extremely important to maintain low filling pressures in the left atrium and in the pulmonary veins, which allows adequate growth of the pulmonary vasculature. Characteristics of blood flow shunting through the foramen should be accurately investigated, taking into account the morphology of the membrane of the foramen and the Doppler signal through it. When blood flow shunts freely from left to right, the membrane appears to flap towards the right, and pulsed Doppler recording shows low velocity (20–40 cm/s) and biphasic flow. Conversely, restrictive foramen ovale appears as a tense, right-convex-shaped membrane, while the flow pattern shows aliasing on color flow mapping with continuous systo-diastolic and high-velocity spectral signal. Moreover, interrogation of the pulmonary veins flow may detect significant flow reversal during atrial contraction, revealing high filling pressure in the left atrium [5]. A restrictive foramen ovale during fetal life in HLHS has important implications as concerns post-natal management and the outcome of the newborn; in fact, it requires prompt intervention at birth to relieve flow obstruction, but afterward, pulmonary vasculature may have been irreversibly damaged by the long-standing low flow and high pressures during gestation.

Careful evaluation of the width and flow pattern across the ductus arteriosus is crucial, being the ductus is the only pathway for systemic output. For this reason, ductal constriction is never observed in viable fetuses with HLHS, and ductal size is rather wide (Figure 1). The augmented flow across the ductus is expressed by a slightly altered Doppler profile, showing a higher pulsatility index (systolic to diastolic flow peak velocity ratio) due to increased systolic with the preserved diastolic flow. Conversely, when the middle cerebral artery is explored, the flow pattern shows a lower pulsatility index with significant diastolic flow due to a protective mechanism of decreased cerebral vascular resistance, called “cephalization” of flow. In fact, because brain perfusion occurs through the duct and the aortic arch in a retrograde fashion, and systolic flow alone may not be sufficient for its oxygen supply, lower vascular resistances ensure adequate cerebral perfusion during diastole [21]. For this reason, there is concern about the neurodevelopmental outcome of newborns with HLHS, and congenital brain anomalies are often encountered, either related to impaired prenatal brain perfusion or in the setting of commonly associated genetic syndromes.

Right ventricular (RV) function is another key point of fetal evaluation. Since RV is the only pumping chamber, any cause of RV dysfunction or overload may not be hemodynamically tolerated and should be carefully investigated [23]. However, quantitative assessment of RV function in utero may not be feasible, and therefore its evaluation is mostly qualitative. Particularly, the tricuspid valve may present some morphologic abnormality, like dysplasia of the leaflets and subvalvular apparatus; hence, the appearance of worsening tricuspid regurgitation in serial examinations should raise a concern about neonatal outcomes (Figure 1).

Coronary artery origin and course are usually normal in HLHS. However, ventriculo-arterial connections, also called sinusoids, are relatively common in the MS/AA variant, though they are not necessarily associated with the worst outcome [24].

Finally, fetal echocardiography has a major role when a transcatheter intervention is planned during fetal life, as it is performed in a limited number of institutions. In this setting, prenatal echocardiography is essential to identify those patients who may ultimately benefit from such intervention, and for instance, these challenging procedures, like aortic balloon dilatation or atrial septostomy, are entirely guided by ultrasounds [25].

## 3. At Birth

Once the newborn is stabilized and prostaglandin infusion has been initiated to maintain ductal patency, echocardiography is expected to define anatomic details and early adaptation from fetal to postnatal physiology. Diagnostic cardiac catheterization at birth is not mandatory; therefore, echocardiography is requested to provide hemodynamic information along with an anatomic assessment.

In the last decades, the hybrid palliation of HLHS has gained favor as an alternative to the classical Norwood operation [1,2]. Targets of palliation are common to both approaches: (1) to provide adequate systemic output from the single ventricle, (2) to balance pulmonary blood flow avoiding pulmonary over circulation, and (3) to allow unrestricted pulmonary venous return to the right atrium. Hence, with the hybrid stage I, these targets are addressed, respectively, by (1) transcatheter stenting of the ductus arteriosus, (2) bilateral banding of the pulmonary artery branches, and (3) stenting of the ASD, when needed.

**Figure 2:**
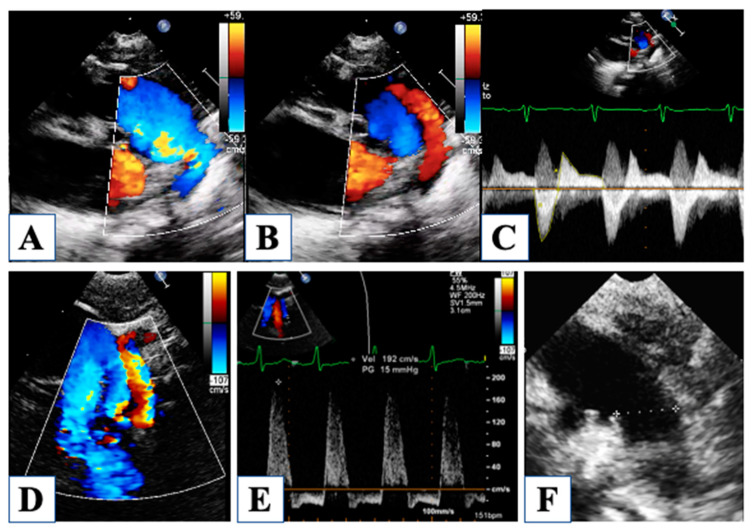
Dilated pulmonary trunk with aortic valve atresia and wide ductus arteriosus providing systemic cardiac output with right-to-left shunt in systole (panel (**A**)) and left-to-right shunt due to lower pulmonary compared to systemic resistances (panel (**B**)). Continous-wave Doppler through the ductus allows measurement of the amount of flow in each direction by tracing the Doppler curves (Panel (**C**)). Panel (**D**) shows a diminutive takeoff of the aortic arch from the ductus, generating increased velocity of the reversed aortic flow measured by Pulsed-wave Doppler (Panel (**E**)). Ductus arteriosus can be very large compared to pulmonary arteries, as shown in panel (**F**).

For instance, the ductal-dependent nature of this disease makes the evaluation of the ductus arteriosus a key point immediately after birth. Parasternal short axis, suprasternal, and the in-between off-axis views allow clear visualization of ductal width, morphology, and flow pattern (Figure 2).

Although the exact measurement of ductal width and length is better obtained by angiography, later in the hybrid suite, a proper echocardiographic assessment of these critical measures can be useful in planning the procedure. In fact, a very short and wide duct may not be suitable for stenting or may carry a high risk of stent migration [6].

**Figure 3:**
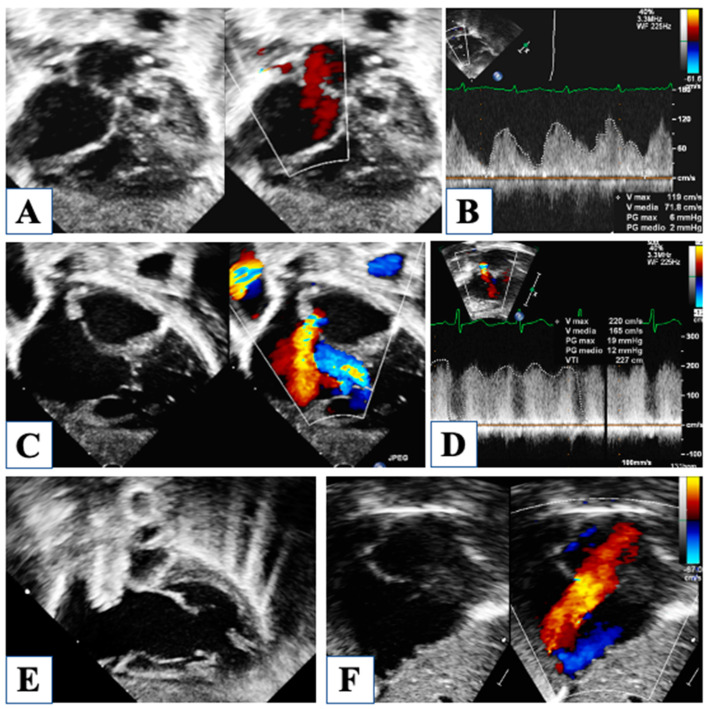
Atrial septal defect with malaligned septum primum and no restrictive flow (Panel (**A**)). Continous wave Doppler showing a low mean gradient of 2 mmHg through the atrial septum (Panel (**B**)). Unrestrictive ostium secundum atrial septal defect (Panel (**C**)). Critically small atrial septal communication with aliasing by color-Doppler (Panel (**D**)) and high mean gradient of 12 mmHg (Panel (**E**)). Successful stenting of the restrictive atrial defect (Panel (**F**)).

Then, at birth, patency of the foramen ovale is critical for neonatal survival, and five different morphologies of the atrial septal defect (ASD) have been described in HLHS [7]. The most common are (1) ostium secundum ASD, followed by (2) malaligned ASD with a posterior and leftward deviation of the septum primum, (3) ostium primum ASD, and less common (4) congenitally small or absent ASD and (5) aneurysm of the septum primum. Restrictive physiology occurs when ASD is small, or sometimes in the case of malaligned ASD, due to the small left atrial size and the unfavorable shape of the defect (Figure 3).

This particular variant of malaligned ASD is characterized by leftward attachment of the posterosuperior aspect of the septum primum, far to the left from the normal central attachment of the septum secundum, therefore creating potential flow obstruction. When this morphology of the ASD with restrictive physiology is observed, and ASD stenting is required, the interventional cardiologist should be alerted of the risk of stent migration due to the irregular, unfavorable shape of the defect. Partial anomalous drainage of the right pulmonary veins has been reported in association with this type of malaligned ASD, even in the setting of normal connection; in fact, right pulmonary veins may still be connected to the leftward aspect of the septum secundum, even draining into the right atrium due to the malaligned septum primum. Furthermore, despite normal anatomic connection, total anomalous pulmonary venous drainage through a persistent vertical vein is described and should be investigated in the presence of a nearly intact ASD [8].

**Figure 4:**
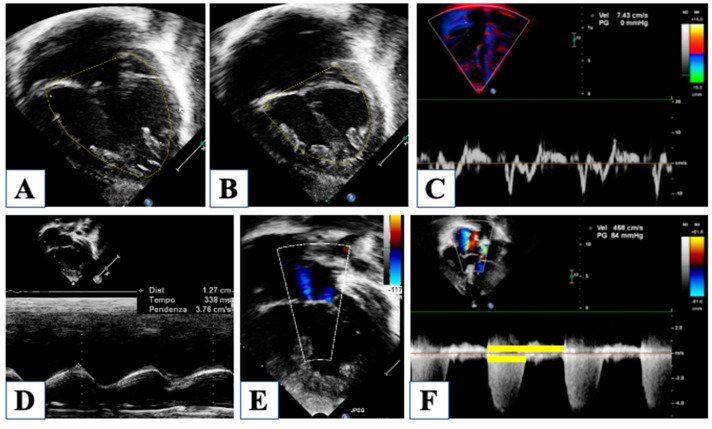
Evaluation of right ventricular function. Fractional area change of the right ventricle is obtained by tracing the endocardial border in diastole (panel (**A**)) and in systole (panel (**B**)). Tissue Doppler imaging of the lateral tricuspid annulus allows the measurement of the annular systolic velocity (panel (**C**)). Tricuspid annular plane systolic excursion is obtained by M-Mode (Panel (**D**)). Mild tricuspid regurgitation (panel (**E**)). Continous-wave Doppler of the tricuspid regurgitation jet allows measurement of the systolic to diastolic duration ratio (Panel (**F**)).

With an underdeveloped LV, the RV is responsible for the total cardiac output, and therefore it is subjected to significantly higher pressure and volume load regimens compared to normal. Furthermore, until stage II with cavo-pulmonary connection is established, the single ventricle is forced to manage more than one stroke volume at the same time, depending on the Qp/Qs. Indeed, RV function is a central issue in the global assessment of a newborn with HLHS, being closely related to long-term outcomes [26]. However, the accuracy in the evaluation of RV function is extremely debated, basically due to the RV complex geometry and tridimensional extent. Furthermore, the unpredictable contribution of the hypoplastic LV to the global ventricular shape can further interfere with RV evaluation. The current guidelines by the American Society of Echocardiography [27] recommend assessing RV global function using RV fractional area change (FAC) and longitudinal function by tricuspid annular plane systolic excursion (TAPSE) (Figure 4).

However, two-dimensional (2D) derived RV volumes result systematically in an underestimated value when compared to those obtained by magnetic resonance imaging (MRI), which represents the gold standard for cardiac volume quantification. Nonetheless, three-dimensional echocardiography has been shown to almost reach MRI accuracy for the assessment of volumes, mass, and ejection fraction, even in the setting of a functional single ventricle [28,29]. However, in clinical practice, neither a 3D echocardiogram nor MRI is often feasible, and the estimation of RV function is qualitative and subjective most of the time. Although suboptimal and not recommended, it should be noted that subjective visual evaluation of RV function, when judged by an experienced operator, forereaches MRI assessment with good sensitivity and specificity [9].

A semi-quantitative method for RV evaluation has been proposed to overcome geometric-derived limitations, and it is based on CW-Doppler tricuspid flow. The systolic to diastolic duration ratio (S/D) is a simple measure of the TR jet duration, which represent the RV systolic interval divided by the interval free from the TR jet, which is occupied by diastolic RV inflow (Figure 4). When S/D duration was tested on HLHS patients, it showed a good correlation with visually and quantitatively assessed RV systolic function [10], with the highest values in HLHS patients with depressed contractility. Although the S/D duration did not correlate as well with invasive end-diastolic pressure, it expresses a non-disease-specific index of systolic and even more diastolic dysfunction, providing a visual assessment of shortened diastolic time intervals. Evaluation of RV function and TR is a central point since they both have a strong impact on survival in this population [11].

Experiences with tissue Doppler velocity and myocardial deformation in HLHS patients have been reported [12,13,14,15] and showed how new modalities might overcome geometrical, angle-dependent, and operator-dependent limitations, providing reliable quantification of regional and global RV function. Moreover, RV mechanical dyssynchrony has been investigated in children with HLHS, and it appears to be related to RV dysfunction and significant tricuspid regurgitation [10]. However, published studies dealt with the classic Norwood approach to HLHS, and therefore, their results cannot be directly applied to patients after hybrid stage I palliation. One reason for this could be the lack of cardiopulmonary bypass with deep hypothermia in the hybrid approach, although their negative impact on post-operative RV dysfunction and remodeling has not been demonstrated [30].

## 4. Morphological Insights: The Borderline LV

**Figure 5:**
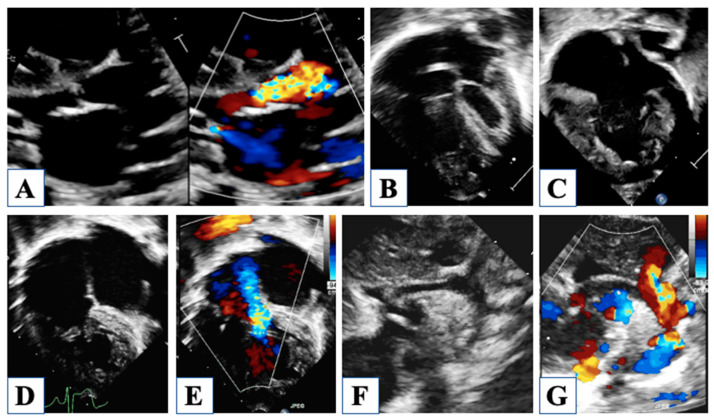
Severe aortic valve stenosis is shown in panel (**A**) from a parasternal long-axis view. Severe mitral valve hypoplasia with evident endocardial fibroelastosis of the left ventricle in panel (**B**). Four-chamber view in panel (**C**) shows a double inlet right ventricle with virtually absent left ventricle. Mitral atresia in panel (**D**,**E**) is associated with dysplasia of the tricuspid valve, with moderate regurgitation. Aortic valve atresia with severely hypoplastic ascending aorta is shown in panel (**F**). Panel (**G**) shows aortic arch hypoplasia with ductal dependency and reverse flow in the aortic arch.

Under the same denomination of HLHS encompassed a spectrum of anatomical variants of left-sided obstructive lesions that share the common feature of an underdeveloped LV, which is unable to support systemic circulation (Figure 5) [31].

In the original description by Lev [32], only the severe end of the spectrum was included, in the forms of MA/AA, MS/AA, and MS/AS, with an intact ventricular septum and variable degrees of aortic arch hypoplasia. Less severe variants of the spectrum are often associated with borderline characteristics of the LV and include critical aortic stenosis, Shone complex, and severe aortic coarctation. Finally, mild LV hypoplasia may occur in association with isolated aortic coarctation, cor triatriatum, or mitral stenosis. The different nomenclature of hypoplastic left heart complex (HLHC) has been recently used to define hypoplasia of the left heart structures with no intrinsic valvular stenosis and typically aortic arch anomalies, usually associated with ventricular septal defect [33].

Beyond the aforementioned HLHS and HLHC variants, there are several situations in which the systemic ventricle is functionally unable to support circulation, like an unbalanced atrio-ventricular septal defect or total anomalous pulmonary venous return, mainly due to RV pressure and/or volume overload resulting in LV compression. However, in case of total anomalous pulmonary venous return or isolated aortic coarctation, the LV is usually adequate to manage systemic circulation after repair, even with preoperative volume as low as 8 mL/m^2^. Moreover, ventriculo-arterial discordance with hypoplastic systemic ventricles or univentricular hearts with aortic outflow obstruction are not accepted in the usual classification of HLHS, but still, they share with HLHS a similar pathophysiology.

With the advent of the hybrid palliation for standard HLHS, followed by single ventricle staged reconstruction, it appeared soon that such a palliative strategy could also be extended to “HLHS-like” situations and even used as a bridge to biventricular repair in all those borderline cases in which the LV would have shown a sufficient growth at the end of the interstage period [16].

A small LV can be referred to as “borderline” when its actual size appears inadequate to sustain systemic output, but it is not excessively undersized to show potential growth after the optimal hemodynamic status has been restored. Furthermore, it has been observed that the timing of the decision plays a crucial role in the fate of the hypoplastic borderline LV because the longer the ventricle is allowed to grow, the higher the probability of achieving functional independence. Hence, the goal of hybrid stage I would be to postpone the decision until LV fully expresses its growth potential; then, many more biventricular repairs might be reached than at the time of the first evaluation.

In this setting, the most accurate assessment of LV size and aortic and mitral valve annular diameter acquires great importance in order to choose the best surgical option. Serial measurements of LV diameters, volumes, and mass, along with mitral and aortic valve annular diameters, LV long axis to heart long axis ratio, and aortic arch diameters, should be carefully reported and expressed as a z score. A standardized assessment, based on currently accepted guidelines for chamber quantification [27], is crucial in the effort to assign each borderline LV to its correct pathway.

Unfortunately, there is no consensus regarding the criteria of the appropriateness of biventricular repair for borderline HLHS instead of what is available for critical aortic stenosis [34,35]. The long-standing dispute about the appropriate way to address a borderline LV is still a matter of debate. Although many indexes and cut-off values have been proposed to orient between biventricular or single-ventricle reconstruction, the borderline left ventricle often forces the heart team to a patient-tailored approach.

## 5. After Hybrid Procedure: The Interstage Period

While echocardiographic features of HLHS after Norwood stage I operation have been widely described, few data are available in the literature about the role of echocardiography in the interstage period after hybrid palliation. The interstage period should last 4 to 6 months, between the hybrid procedure and the subsequent “comprehensive stage II operation”, which consists of stent removal, aortic arch reconstruction and anastomosis with main pulmonary artery, debanding and augmentation of pulmonary artery branches with superior cavo-pulmonary anastomosis, and atrioseptectomy. The interstage period has been recognized to be a very frail phase in which the hemodynamic balance is highly labile and needs close surveillance. In this setting, echocardiography, along with clinical evaluation, plays a key role in the tight follow-up that these particular patients require.

**Figure 6:**
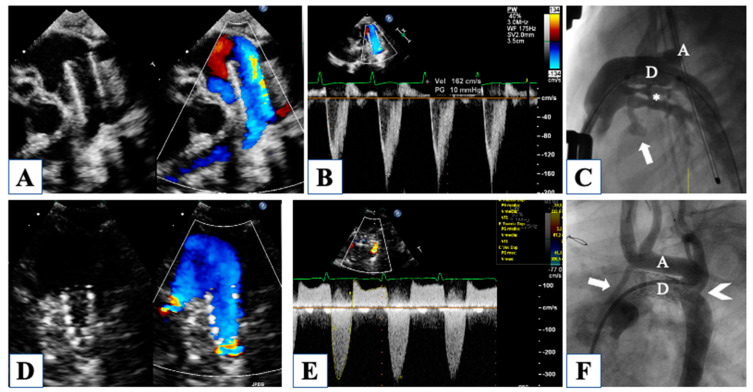
Hybrid stage I palliation. In panel (**A**), the ductal stent is well expanded, and color Doppler shows normal flow velocity through the stent, as confirmed by pulsed-wave Doppler in Panel (**B**). In Panel (**C**), the angiogram delineates clearly the ductal arch (D), the banded pulmonary arteries (*), and the retrograde perfusion of the aortic arch (A) with severely hypoplastic aortic valve (arrow). In panel (**D**), an example of obstruction at the distal end of the ductal stent, as evidenced by the aliasing (similar for the stent and for the banded pulmonary branch on the left) and by the continuous wave Doppler in Panel (**E**). Panel (**F**) shows a potential mechanism of distal obstruction of the stent, with prominent posterior aspect of the aortic isthmus (arrowhead); the arrow is pointing to the severely hypoplastic ascending aorta, while transverse aortic arch has normal diameter (A); ductal stent is well visible (D).

For instance, ductal stents must be carefully evaluated from suprasternal and parasternal views to ensure that there is no obstruction to forward flow through the stent (Figure 6).

Different types of stents have been reported for ductal stenting (balloon-expandable or self-expandable stents), but all share the same potential complications in terms of lumen obstruction, which can occur in a central position or at the proximal or distal edge [6,36]. Color flow mapping and continuous wave (CW) Doppler are able to identify any obstruction within the stent, although a precise localization of the site of restriction may not be possible. A peak velocity of forward ductal flow higher than 1.7 m/s, which corresponds roughly to a 12-mmHg pressure gradient, has been shown to predict repeat interstage intervention [17].

A serious complication that can occur in up to 29% of cases during interstage is a retrograde aortic arch obstruction (Figure 2) [18]. Since cerebral and coronary perfusion is mainly, if not entirely, dependent on retrograde flow through the aortic arch, a “reverse coarctation” has a strong impact on morbidity and mortality. Color Doppler echocardiography shows flow turbulence at the aortic isthmus and CW–Doppler can reliably estimate the pressure gradient through the obstruction [18]. Systolic peak velocities of obstructed retrograde aortic flow can be as high as 3 m/s, and the decision to intervene is based on clinical signs of reduced cerebral and coronary perfusion. ST-segment elevation on electrocardiogram and evidence of decreasing RV function with concomitant increasing tricuspid regurgitation should immediately orient the observer towards the presence of reverse coarctation. It should be noted that the negative impact of retrograde aortic obstruction can vary with the anatomical variants of HLHS. In the case of preserved, though small, aortic forward flow, the effect of reverse coarctation is expected to have a less detrimental effect than it has for aortic atresia [18]. Consequently, it has been postulated that the diameter of the aortic root, as measured from the parasternal long axis view and expressed as a z-score, can be considered as a risk factor for reverse coarctation, with risk increasing proportionately to negative z-score [18].

In the ideal system with aortic atresia and negligible pulmonary regurgitation, the total cardiac output can be calculated from the antegrade velocity-time integral (VTI) on the CW–Doppler curve through the ductal stent (Figure 6). However, a retrograde flow during diastole can also be observed, whose entity can be extremely variable. Calculation of the retrograde VTI accounts for the amount of diastolic pulmonary flow, which is “stolen” from the systemic output. Consequently, systemic cardiac output can be assumed to be the difference between antegrade and retrograde VTI, based on the continuity equation and considering the narrowest ductal diameter to calculate it [19]. The difference between ductal antegrade (total cardiac output) and ductal retrograde flow has been referred to as “effective flow”, which allows perfusion of the retrograde aortic arch and descending aorta. In the presence of aortic stenosis with a source of forward aortic flow, this assumption cannot be applied since its accuracy would be inversely proportional to the entity of forward aortic flow. A small ductal reverse VTI has been related to better outcomes after comprehensive stage II in terms of better systemic cardiac output, expressed by improved urine output and lower lactate levels post-operatively.

**Figure 7:**
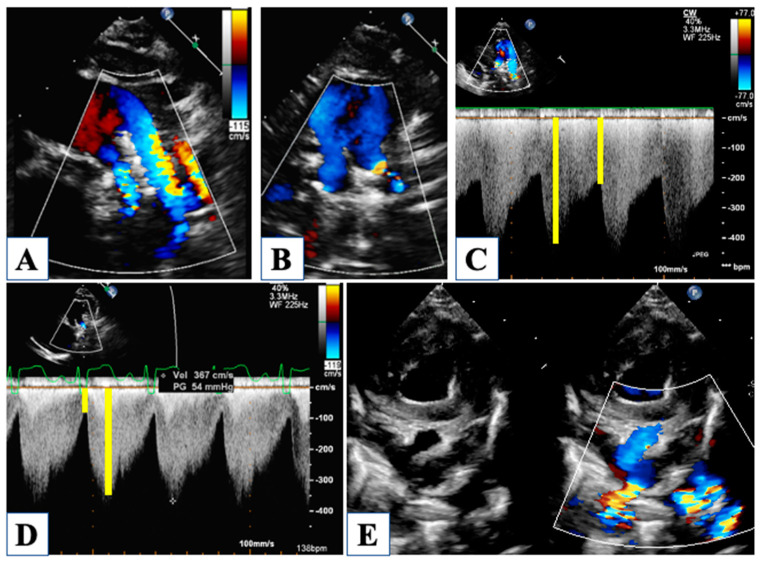
Pulmonary branches banding. The right pulmonary artery is well visible below the ductal stent in Panel (**A**), while both branches may be imaged at same time from a high parasternal short axis (Panel (**B**,**E**)) with evident aliasing on color Doppler. Continuous-wave Doppler through the banded branches shows sisto-diastolic high-velocity flow, in panel (**C**) a normal Doppler pattern, in panel (**D**) an example of high pulsatility index (high systolic velocity with low diastolic velocity) and steep systolic slope consistent with high pulmonary pressures.

In hybrid stage I palliation, pulmonary blood flow is provided by the native pulmonary arteries which are selectively narrowed with Goretex bands to avoid pulmonary overload. This might appear as a technically easy procedure, but the subtle equilibrium between excessive and insufficient narrowing that may inadvertently lead to pulmonary hypoperfusion or over-circulation makes this surgical gesture actually challenging [37]. Moreover, the entity of restriction needs to be tailored to any different patient due to the individual variability of pulmonary vascular resistances. Thus, interstage echocardiographic surveillance of pulmonary arteries flow has become crucial (Figure 7).

Though anatomical distortion of the banded pulmonary arteries can cause difficult visualization from the usual views, CW–Doppler through the narrowed site usually provides a reliable measure of the amount of flow that is distributed to the lungs. For this purpose, a certain number of Doppler parameters have been studied that can help with noninvasive hemodynamic assessment during interstage. Systolic and diastolic peak velocities should be noted and related to each other to express the pulsatility index. Moreover, velocity-time integral, systolic slope, and pressure half-time of the waveform is useful to better characterize pulmonary flow pattern. When high systolic peak velocity is observed along with low diastolic velocity, the pulsatility index increases, and this can be translated into elevated pulmonary pressure. A steeper slope with reduced pressure half-time indicates rapid equalization of pressures across the bands, and this phenomenon has been observed in association with ASD restriction, which is responsible for increased diastolic pulmonary pressure, counteracting flow access through the bands. This concept has been documented by the “normalization” of the pulmonary flow Doppler waveform after relief of ASD restriction; in fact, with reduced diastolic pressure in the pulmonary vascular bed, the pulsatility index and pressure half-time increased, along with a less-steep slope of the waveform [17].

Because the natural tendency of ASD is towards spontaneous restriction, it is important to ensure that pulmonary venous return to the right atrium is kept unrestrained. Even more, in the presence of a stented ASD, tight surveillance is required. Along with direct signs of turbulent flow through the ASD, usually well seen with color and CW-Doppler, it can be useful to look for indirect signs of high left atrial end-diastolic pressure, as reported about modification of the pulmonary flow waveform across the bands [17].

It should be underlined that all these measurements do not assume absolute value, but they should rather be considered as dynamic values, expected to change over time. Several reasons can be mentioned to explain these changes. First of all, during the interstage, the patient is expected to grow up as much as possible, even reaching twice or more his/her initial weight. Likewise, the heart and vessels grow up, but non-native tissues do not, like ductal stents and pulmonary bands. Then, pulmonary vascular resistances tend to decrease over time, making the initially adequate banding inappropriate. Finally, the interaction of native tissues with the ductal stent leads to cellular proliferation, so progressive lumen obstruction is not a rare complication. Particularly, the takeoff of the retrograde aortic arch is a matter of concern because whatever cause of impaired flow (reverse coarctation, stent displacement, steal phenomenon) can translate into life-threatening central hypoperfusion. For these reasons, all hemodynamic parameters that can be assessed by echocardiography should be carefully reported during interstage evaluations and compared with previous recordings. Peak flow velocities through the ductal stent, retrograde aortic arch, pulmonary bands, and ASD are all expected to increase over time, so the actual challenge is to detect how much change is too much. So far, the answer cannot be achieved by echocardiography alone, but a high-grade suspicion in the setting of a thorough clinical evaluation is needed.

## 6. After Comprehensive Stage 2 Operation

The term “comprehensive” has been coined to define the incorporation of surgical aspects pertaining to the classical Norwood stage I and II. The optimal timing of this second-stage palliation after the hybrid approach is not known since it may not necessarily be the same as for classic Norwood, for which the best long-term outcome has been identified in patients who received the second-stage operation between 3 and 6 months [38]. Aortic arch reconstruction and amalgamation with the main pulmonary artery and atrioseptectomy, which are part of stage I, and superior cavo-pulmonary anastomosis that characterizes stage II are performed after stent removal and debanding with subsequent augmentation of pulmonary artery branches. Consequently, echocardiographic features could be considered similar to those already described for post-operative Norwood stage I, with a remarkable difference in terms of hemodynamic profile. In fact, the source of pulmonary blood flow is no longer a systemic-to-pulmonary shunt, which steals blood from the neo-aortic flow, but rather a bidirectional cavo-pulmonary anastomosis.

**Figure 8:**
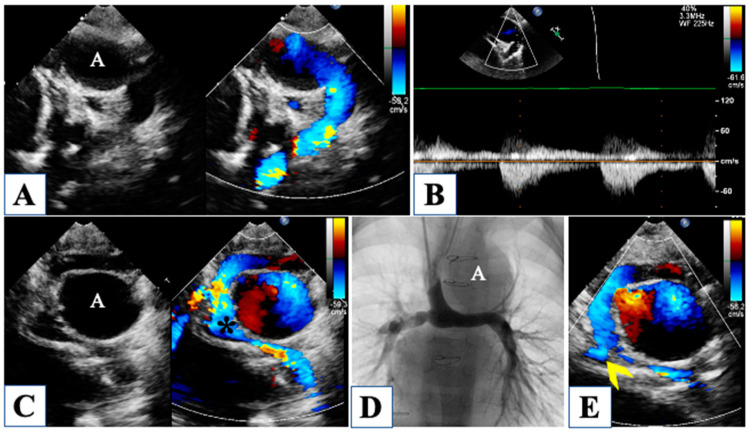
Prominent aortic arch reconstruction with dilatation of the transverse arch (A), compared to the normal size of the descending aorta, without Doppler signs of obstruction (Panel (**A**,**B**)). In panel (**C**), the superior cavo-pulmonary anastomosis (*) with partial compression of the pulmonary branches. Angiography of the pulmonary branches (Panel (**D**)) shows significant stenosis of the right pulmonary artery, whose ostium (arrowhead) is not easily visualized by echocardiography (Panel (**E**)).

The surgically reconstructed neo-aorta must be carefully investigated to rule out any obstruction, particularly at the distal site of anastomosis (Figure 8).

In addition to a Doppler peak gradient higher than 30 mmHg through the distal arch, a useful parameter has been proposed to detect distal obstruction after Norwood stage I, that is, the “coarctation index”. It is defined as the ratio of the narrowest to the widest descending aortic diameter, and a value below 0.7 has been correlated with coarctation [20]. Since after Norwood stage I, the Blalock-Taussig shunt leads to diastolic reversal flow in the aortic arch, a hypothetical aortic coarctation would not produce diastolic runoff, as it would be counterbalanced by a diastolic steal in the shunt; therefore the “coarctation index” would detect more accurately an obstruction, being based on morphology rather than on Doppler assessment. Moreover, the surgical arch reconstruction may impact ventriculoatrial coupling, thus increasing RV global afterload [39].

A critical evaluation of the atrial septum is warranted whenever clinical observation of cyanosis would raise the suspicion of inadequate atrial septal resection. However, increased velocities might also be due to augmented pulmonary venous return as it occurs in the case of pulmonary overcirculation.

Perhaps the reconstruction of the pulmonary artery branches represents Achille’s heel of the whole hybrid palliation strategy. Comparing the hybrid palliation to the classic Norwood strategy for HLHS, a substantial equivalence in terms of survival has been observed, but it has been underlined that the hybrid approach carries a higher rate of pulmonary artery branches stenosis, requiring repeat reintervention (Figure 8) [40,41]. In fact, the patch plasty of the pulmonary branches after debanding frequently leads to proximal to distal branch mismatch, resulting in flow obstruction. Moreover, the left pulmonary artery (which is actually the previous right native pulmonary artery before the bidirectional Glenn anastomosis) courses behind the neo-aorta, and because it is a flexible, reconstructed structure, it can easily be flattened and compressed. However, echocardiography is not always able to image pulmonary arteries, and not infrequently; it fails to detect pulmonary branch stenosis [42]. For this reason, a diagnostic cardiac catheterization 2 to 4 months after comprehensive stage II has been advocated to accurately rule out any residual or new onset pulmonary flow obstruction.

RV function should be carefully evaluated with serial monitoring, along with surveillance of tricuspid regurgitation and neo-aortic regurgitation.

## 7. Conclusions

In the last two decades, a hybrid approach has been adopted by many institutions as a possible palliative strategy for patients with HLHS. Echocardiography plays a crucial role throughout the whole management of hybrid palliation and should be evaluated by highly skilled operators. First, ultrasound techniques are a prerequisite for the diagnosis and evaluation of candidacy for hybrid procedures. Second, during interstage echocardiographic surveillance of life-threatening complications is warranted. Finally, after comprehensive stage II, close monitoring by imaging is still required to ensure a favorable outcome for such complex and frail patients.

## Figures and Tables

**Table 1:** List of publications reporting the role of echocardiography in different stages of palliation for hypoplastic left heart syndrome.

Author	Ref. Number	Stage	Echo Parameter	Measurement Required	Meaning
Michelfelder et al., 2005	[5]	Fetal	S/D velocity and forward/reverse VTI ratio	Pulmonary venous flow pattern	Increased reverse flow correlated with restrictive ASD
Boucek et al., 2015	[6]	Pre-stage I	Ductal anatomy	Orientation, length of the ductus	Suitability for stenting
Chin et al., 1990	[7]	Pre-stage I Interstage Stage II	Atrial septum morphology	Atrial septal communication	Implications on interventional/surgical management
Seliem et al., 1992	[8]	Pre-stage I	Connection and drainage of pulmonary veins	Bidimensional, Color flow Doppler of pulmonary veins	Identification of anomalous pulmonary venous drainage
Bellsham-Revell et al., 2013	[9]	Pre-stage I	RV function	Qualitative assessment (validated against magnetic resonance)	Impact of RV dysfunction on survival
Friedberg et al., 2007	[10]	All	S/D duration ratio	Tricuspid regurgitation jet	High S/D ratio correlated with worse RV function
Carrillo et al., 2021	[11]	All	RVFAC, TAPSE, Tricuspid regurgitation	RV area change, annular excursion, tricuspid regurgitation jet (vena contracta)	Impact on survival at all stages
Petko et al., 2011	[12]	After stage I	RVFAC, TAPSE, Tricuspid regurgitation	RV area change, annular excursion, tricuspid regurgitation jet (vena contracta)	Impact on survival at all stages
Bellsham-Revell et al., 2012	[13]	All stages (Norwood)	TDI indices, Myocardial performance index, S/D duration ratio	TDI of the RV free wall, tricuspid regurgitation jet	Myocardial performance index increased compared to normal values.
Tham et al., 2014	[14]	All stages (Norwood)	Longitudinal and circumferential deformation	Speckle tracking-derived longitudinal and circumferential strain and strain rate	Inability of the single RV to fully adapt to systemic pressures.
Michielon et al., 2020	[15]	All stages (Norwood)	RVFAC, TAPSE, longitudinal deformation	RV area, annular excursion, speckle tracking-derived longitudinal strain, and strain rate	Impact on outcome at all stages
Oreto et al., 2023	[16]	Pre-stage I	Left ventricle, aortic and mitral valves, long axis ratio	Aortic annulus, mitral annulus, aortic root, left ventricular/heart long axis, left ventricular volumes, and mass	Evaluation of the borderline left ventricle
Fenstermaker et al., 2008	[17]	Interstage I–II	Pulmonary arteries flow velocity, pulsatility index, and systolic/diastolic ratio;	CW Doppler on pulmonary arteries	Correlation with ASD restriction, ductal obstruction, aortic arch obstruction
			ASD mean gradient, ductus arteriosus peak velocity, retro-aortic arch peak velocity	CW Doppler through ASD, ductus and aortic arch	
Egan et al., 2011	[18]	Interstage I–II	Smaller aortic root, higher flow velocity from the retrograde aortic arch	Linear measurement of aortic root, CW Doppler through the retrograde aortic arch	Prediction of retrograde aortic arch obstruction
Birnbaum et al., 2010	[19]	Interstage-stage II	Retrograde ductal VTI, RV FAC	CW Doppler through the ductus, RV area change	Correlation with peri-operative cardiac output
Lemler et al., 2000	[20]	After Norwood stage I	Coarctation index	Narrowest/widest descending aortic diameter ratio	A value < 0.7 correlates with coarctation

Abbreviations: S/D systolic/diastolic, VTI velocity time integral, ASD atrial septal defect, RV right ventricular, RVFAC right ventricular fractional area change, TAPSE tricuspid annular plane systolic excursion, TDI tissue Doppler imaging.

## Data Availability

Data is contained within the article.
